# Diagnostic value of HE4+ circulating tumor cells in patients with suspicious ovarian cancer

**DOI:** 10.18632/oncotarget.23943

**Published:** 2018-01-04

**Authors:** Yan-Xiu Guo, Kuang Hong Neoh, Xiao-Hong Chang, Yukun Sun, Hong-Yan Cheng, Xue Ye, Rui-Qiong Ma, Ray P.S. Han, Heng Cui

**Affiliations:** ^1^ Center of Gynecologic Oncology, Peking University People’s Hospital, Beijing, China; ^2^ College of Engineering, Peking University, Beijing, China

**Keywords:** circulating tumor cells, microfluidic isolation, HE4, ovarian cancer, diagnosis

## Abstract

Lacking a satisfactory screening test, ovarian cancer is frequently diagnosed at a late stage, leading to poor patient outcomes. This study investigated the diagnostic value of circulating tumor cells (CTCs) in peripheral blood from patients with suspected ovarian tumors. Sixty-one women suspected of having an ovarian mass were prospectively enrolled in this study. CTCs were identified and counted using microfluidic isolation and immunofluorescent staining of CD45, HE4, and epithelial and mesenchymal (E&M) markers (epithelial cell adhesion molecule, cytokeratins, and vimentin). Thirty (49%) of the patients were diagnosed with ovarian cancer. DAPI+/E&M+/CD45-/HE4+ CTC counts were higher in these patients than in patients with benign tumors (*p* = 0.016). The receiver operating characteristic (ROC) curve showed that the sensitivity of CTCs was 73.3%, which was superior to that of CA125 (56.7%). In patients with elevated CA125 levels (≥35 U/ml), CTC counts still showed good specificity (86.7%). Our findings suggest the DAPI+/E&M+/CD45-/HE4+ CTC count is a useful diagnostic indicator in patients with suspected ovarian cancer.

## INTRODUCTION

Ovarian cancer is the fifth leading cause of cancer mortality in women [1], and approximately 521,000 new cases were diagnosed in China in 2015 [2]. Because the symptoms of ovarian cancer are fairly non-specific and there is no satisfactory screening test, about 70% of all ovarian cancer patients are diagnosed at advanced stages [3] with metastases primarily in the peritoneal cavity [4, 5]. These patients usually undergo debulking surgery prior to receiving a combined chemotherapy regimen including a platinum-based drug and taxane [5]. Nevertheless, over 50% of cases eventually relapse [3, 6]. Relapse and metastasis are the major contributors to poor overall survival (OS) rates (<35%) in advanced ovarian cancer patients [7]. Thus, a method for early detection of relapse and/or metastasis would be highly useful for ovarian cancer patient management and care. Epithelial ovarian cancer metastasis is widely believed to occur via direct surface spread through the peritoneal circulation [8, 9]. However, recent research also supports the hematogenous route as an important mode of ovarian cancer omental metastasis through a parabiosis model [10].

In the hematogenous route, circulating tumor cells (CTCs), which have shed from solid tumors and intravasated, can circulate throughout the body and attach at distant organs to grow metastatic lesions. CTCs in peripheral blood have been used as predictive biomarkers for early diagnosis and prognosis of ovarian [4, 6, 7, 11, 12], breast [13], lung [14], and bladder [15] cancers. CTCs enriched by the cell adhesion matrix (CAM) method showed an 83% sensitivity and 95% specificity in detecting ovarian cancer [4]. Various methods have been used to identify and capture CTCs from the peripheral blood, including a physical isolation method that exploits density and/or size differences and an immunoaffinity-based method that targets specific antigen-antibody interactions [16, 17]. Although immunoaffinity-based isolation methods, such as the CellSearch system, have higher capture specificities, some CTC subpopulations may escape detection. Similarly, systems that employ the epithelial cell adhesion molecule (EpCAM), which is the most widely used immunoaffinity antibody, will not identify and capture mesenchymal CTCs that may correlate with metastatic potential [18]. Size-based harvesting methods are more reliable as they are premised on the fact that CTCs (∼10–20 µm) are larger than normal erythrocytes (∼6–8 µm) and leukocytes (∼7–12 µm) [19]. Leukocytes may be captured along with CTCs, but are easily differentiated using CD45. Major CTC detection methods include immunocytochemistry (ICC) [20], reverse-transcription polymerase chain reaction (RT-PCR) [21], flow cytometry [22], and fluorescence *in situ* hybridization (FISH) [16]. Recent work suggests that CTC detection rates in ovarian cancer patients vary widely (12–90%) across different platforms (Table 1) [5, 7, 10, 16, 17, 21, 22, 25–35]. This can be attributed to varying capture efficiencies and detection limits, and the use of specific markers. Additionally, most studies have focused on the prognostic value of CTCs rather than their diagnostic value (Table 1).

**Table 1:** Detection and prognostic relevance of CTCs in ovarian cancer

Authors (year)	Study Type	No. of Patients	Timing	Capture Method	Detection Method	Targeted Antigen/Gene	Positive Rate (%)	Prognostic Significance
Marth *et al.* (2002) [20]	Case Series	90	Before adjuvant chemotherapy	Immunomagnetic (Dynabeads®)	ICC	MOC-31	12.0	NS
Kurata *et al.* (2002) [21]	Case Series	24	Not mentioned	Not separated	RT-PCR	CK7, CK20	46	Not mentioned
Sapi *et al.* (2002) [23]	Case Series	28	Before any therapy	Immunomagnetic	ICC	HEA-125, CD45	75	NS
Judson *et al.* (2003) [24]	Case Series	59	Before surgery	Immunomagnetic beads	ICC	CK8 and 18, TFS-2, CK7, CK20, EGFR	18.7	NS
Oikonomopoulou et al. (2006) [25]	Case Series	24	Before and after therapy	Immunomagnetic separation	RT-PCR	Kallikreins, BER-EP4	75	NS
Wimberger *et al.* (2007) [26]	Case Series	57	57 before therapy & 45 after therapy	Density gradient	ICC	A45-B/B3 , CK8, 18, 19	21	NS
He *et al.* (2008) [22]	Case Series	20	Not mentioned	Density gradient	Flow cytometry	Folate-AlexaFluor 488 & DUPA-FITC	90	NS
Fan *et al.* (2009) [6]	Case Series	66	Before surgery	CAM+ functional enrichment	ICC	EpCAM; CK 4, 5, 6, 8, 10, 13 and 18	60.6	NS
Behbakht *et al.* (2011) [27]	Phase II Clinical	54	Before and after Temsirolimus	Immunomagnetic (CellSearch)	ICC (CellSearch)	EpCAM	44.0 (bef cycle 1)	NS
Poveda *et al.* (2011) [11]	Phase III Clinical	216	Before 2nd line chemotherapy	Immunomagnetic (CellSearch)	ICC (CellSearch)	EpCAM; CK8, 18 and 19	14.4	NS
Aktas *et al.* (2011) [7]	Case Series	122	Before surgery and/or after chemotherapy	Immunomagnetic (Adnatest)	RT-PCR (Adnatest)	EpCAM, MUC-1, CA-125 HER-2	19.0 (bef surgery) 27.0 (after CT)	HR = 4.56 (1.94–10.73) *p =* 0.05
Liu *et al.* (2013) [28]	Case Series	30 new 48 recurrent	Before chemotherapy	Immunomagnetic (CellSearch)	ICC (CellSearch)	EpCAM	60.0 new 53.8 recurrent	NS
Obermayr *et al.* (2013) [17]	Case Series	216	Before surgery & after adjuvant chemotherapy	Density gradient centrifugation RNA extraction (Qiacube system)	RT-PCR	PPIC, GPX8, CDH3, TUSC3, COL3A1, LAMB1, MAM, ESRP2, AGR2, BAIAP2L1, TFF1, EpCAM	24.5 (bef surgery) 20.4 (after CT)	HR = 2.3 (1.1–4.8) *p =* 0.024
Pearl *et al.* (2014) [4]	Case Series	88	Before surgery	CAM (functional enrichment)	ICC	EpCAM, CA-125, DPP4 & CKs	88.6	HR = 1.06 (0.41–2.73) *p =* 0.0219
Kuhlmann *et al.* (2014) [12]	Case Series	143	Before surgery	Immunomagnetic (Adnatest)	RT-PCR (Adnatest)	EpCAM, MUC1, MUC6, ERCC1	14.0	HR = 1.85 (1.03–3.32) *p =* 0.041
Ning N *et al.* (2014) [16]	Case Series	141	Before and 7th day after surgery	Immunomagnetic beads-CD45	FISH (centromere probe 8) ICC	CD45, CK	76.2	Not mentioned
Kolostova *et al.* (2015) [29]	Case Series	118	Before surgery	MetaCell	CM/RT-PCR	MUC1, EpCAM, CA125	65.2	NS
Kolostova *et al.* (2016) [30]	Case Series	56	Before surgery	MetaCell	ICC	-	58	Not mentioned
This article	Case Series	30 patients 31 controls	Before surgery	Size-based microfluidic separation	ICC	EpCAM, panCK, CK7, Vimentin and HE4	73.3	

NS: No Significance.

Human epididymis protein 4 (HE4), encoded by the *WFDC2* gene and also known as WAP four-disulfide core domain protein 2 (WFDC2), was first introduced as a potential ovarian cancer biomarker in 2003 [31]. HE4 is overexpressed on the surfaces of epithelial ovarian carcinoma cells, but not on normal ovary cells [32]. Our previous study showed that serum HE4 level had a sensitivity and specificity for ovarian cancer of 73% and 90–100%, respectively [33], and was superior to CA125 (sensitivity, 88%; specificity, 36–99%). We further showed elevated HE4 expression in established ovarian cancer cell lines [29]. We hypothesized that HE4 could be a promising marker for detecting and identifying CTCs and, subsequently, ovarian cancers. The present study established a new, HE4-based immunofluorescence staining strategy using a microfluidic assaying to detect epithelial ovarian cancer CTCs. We found that HE4+ CTCs were more sensitive than CA125 in identifying patients at high risk for ovarian cancer.

## RESULTS

### Determining chip capture efficiency using a cancer cell line

**Figure 1:**
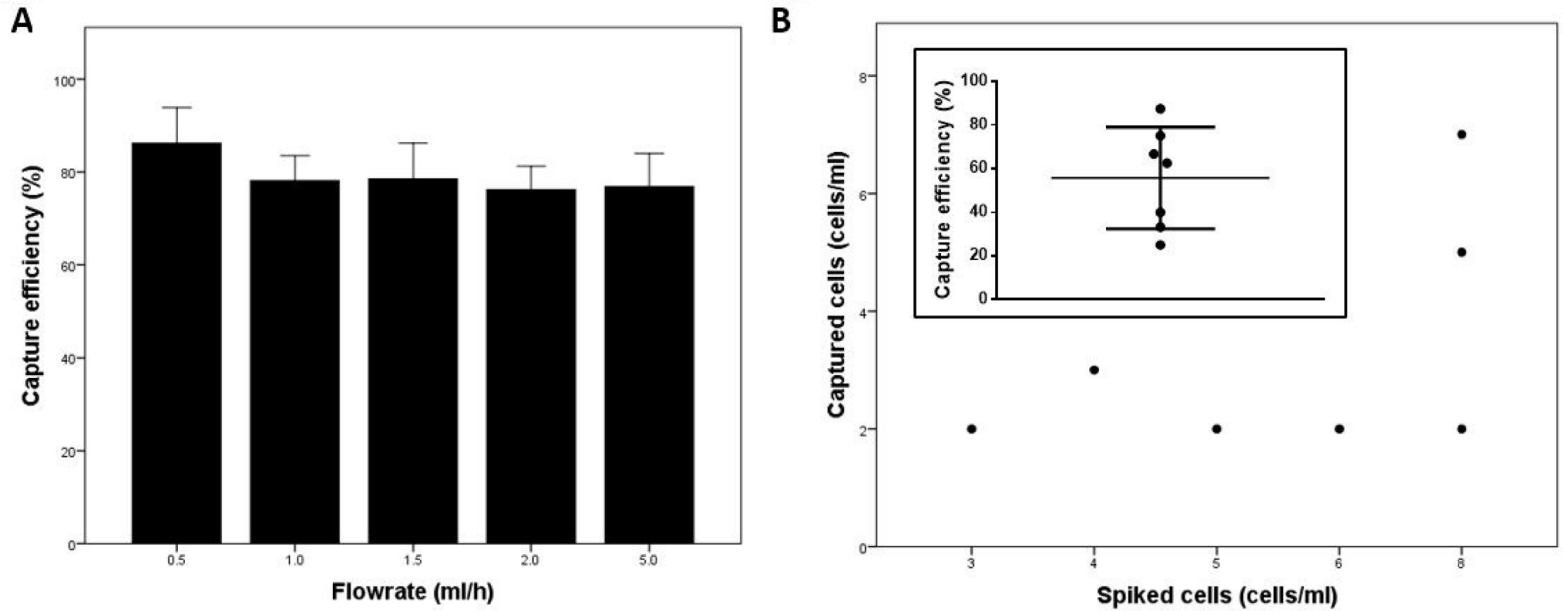
Chip characterization (**A**) Chip capture efficiency at various flowrates (*n =* 3 per flowrate). SKOV3ip1 cells (50 cells/ml) were spiked into healthy donor blood samples. (**B**) Number of cells captured in the chip vs. number of cells spiked into the buffer solution. Inset: Chip capture efficiency using buffer solution spiked with very low numbers of cells (*n =* 7).

To characterize the cancer cell capture efficiency of the microfluidic chip, small numbers of pre-stained SKOV3ip1 cells (50 cells/ml) were spiked into healthy donor blood samples and flowed through the chip. The main factor affecting cell capture efficiency was blood sample flowrate, and capture efficiency decreased with increasing flowrate (Figure 1A). The highest capture efficiency was achieved at 0.5 ml/h (86.2 ± 6.3%). To test the chip’s capture efficiency at ultralow concentrations of cancer cells, <10 SKOV3ip1 cells were spiked into 1 ml of 1% bovine serum albumin (BSA) in 1× phosphate-buffered saline (PBS). We found that the chip’s average capture efficiency was 55.7 ± 23.2% (*n =* 7) (Figure 1B). These results demonstrated that the microfluidic chip efficiently captured cancer cells even at low numbers.

### Patient characteristics

Of the 61 enrolled patients, 30 (49%) were diagnosed with ovarian cancer, 25 (41%) had benign diseases, and the remaining 6 (10%) had other malignant diseases. In the ovarian cancer group, 19 patients were diagnosed with high-grade serous ovarian carcinoma, five with ovarian clear cell carcinoma, two with low-grade serous ovarian carcinoma, two with endometrioid carcinoma, one with immature teratoma, and one with ovarian adult granulosa cell tumor (Supplementary Figure 1). In the benign group, eight patients were diagnosed with serous cystadenoma, four with mucinous cystadenoma, three with Brenner tumor, three with mature teratoma, two with borderline serous cystadenoma, two with borderline mucinous cystadenoma, two with fibroma, and one with ovarian simple cyst (Supplementary Table 2). In the “other malignant” group, two patients were diagnosed with Krukenberg tumor, one with retroperitoneal tumor, one with rectal metastatic carcinoma, one with colon metastatic carcinoma, and one with breast metastatic carcinoma (Supplementary Table 3). Patients diagnosed with ovarian cancer were older than those in the benign group, but patient weights, heights, and body mass indexes (BMI) did not differ significantly between groups (Supplementary Table 4).

Most ovarian cancer patients (53.3%) were diagnosed at International Federation of Gynecology and Obstetrics (FigureO, 2013) Stage III (Table 2). Of all patients who received cytoreductive surgery, two-thirds did not have macroscopic residual disease or lymph node involvement.

**Table 2:** Baseline characteristics of enrolled patients

Total	No. of patients (%)	Captured CTC/mL		
	61 (100)	A cells (DAPI+/E&M+/CD45-/HE4+)	B cells (DAPI+/E&M-/CD45-/HE4+)	C cells (DAPI+/E&M+/CD45-/HE4-)
Tumor stage (Kruskal-Wallis test)		*p =* 0.064	*p =* 0.105	*p =* 0.742
Benign	25 (41.0)	0.8 ± 1.5 (0–6)	0.1 ± 0.6 (0–3)	2.7 ± 6.6 (0–31)
Stage I	8(13.2)	2.1 ± 1.8 (0–6)	0.0 ± 0.0 (0–0)	2.5 ± 3.4 (0–8)
Stage II	4 (6.6)	2.0 ± 2.1 (0–5)	0.3 ± 0.5 (0–1)	2.0 ± 2.4 (0–5)
Stage III	16 (26.4)	2.3 ± 2.5(0–8)	0.06 ± 0.3 (0–1)	2.3 ± 4.2 (0–15)
Stage IV	2 (3.3)	1.0 ± 1.4 (0–2)	0.5 ± 0.71 (0–1)	0.0 ± 0.0 (0)
Other malignant diseases	6 (9.8)	0.5 ± 0.8 (0–2)	0.0 ± 0.0 (0–0)	2.8 ± 4.2 (0–11)
Macroscopic residual disease^*^ (*t*-test)		*p =* 0.741	*p =* 0.364	*p =* 0.776
Yes	10 (34.5)	2.2 ± 2.3 (0–8)	0.1 ± 0.3 (0–1)	2.4 ± 3.2 (0–10)
No	19 (65.5)	2.1 ± 2.2 (0–7)	0.1 ± 0.2 (0–1)	2.2 ± 3.9 (0–15)
Lymph node involvement^*^ (ANOVA)		*p =* 0.53	*p* <0.001	*p =* 0.117
Yes	7 (23.3)	3.0 ± 2.5 (0–7)	0.1 ± 0.4 (0–1)	3.0 ± 5.4 (0–15)
No	17 (56.7)	2.1 ± 2.3 (0–8)	0.0 ± 0.0 (0)	2.4 ± 3.3 (0–10)
Unmeasured†	6 (20)	1.3 ± 1.2 (0–3)	0.3 ± 0.5 (0–1)	0.5 ± 1.2 (0–3)
CA-125 (*t*-test)		*p =* 0.966	*p =* 0.635	*p =* 0.724
≤ 35 U/mL	21 (36.7)	1.5 ± 2.2 (0–8)	0.1 ± 0.7 (0)	1.7 ± 3.4 (0–15)
> 35 U/mL	36 (63.3)	1.5 ± 1.9 (0–7)	0.1 ± 0.3 (0–1)	2.1 ± 3.5 (0–10)

^*^Benign samples were not included for these characteristics.

^†^Did not undergo the surgery or lymph node dissection.

### Patient baseline CTC counts

**Figure 2:**
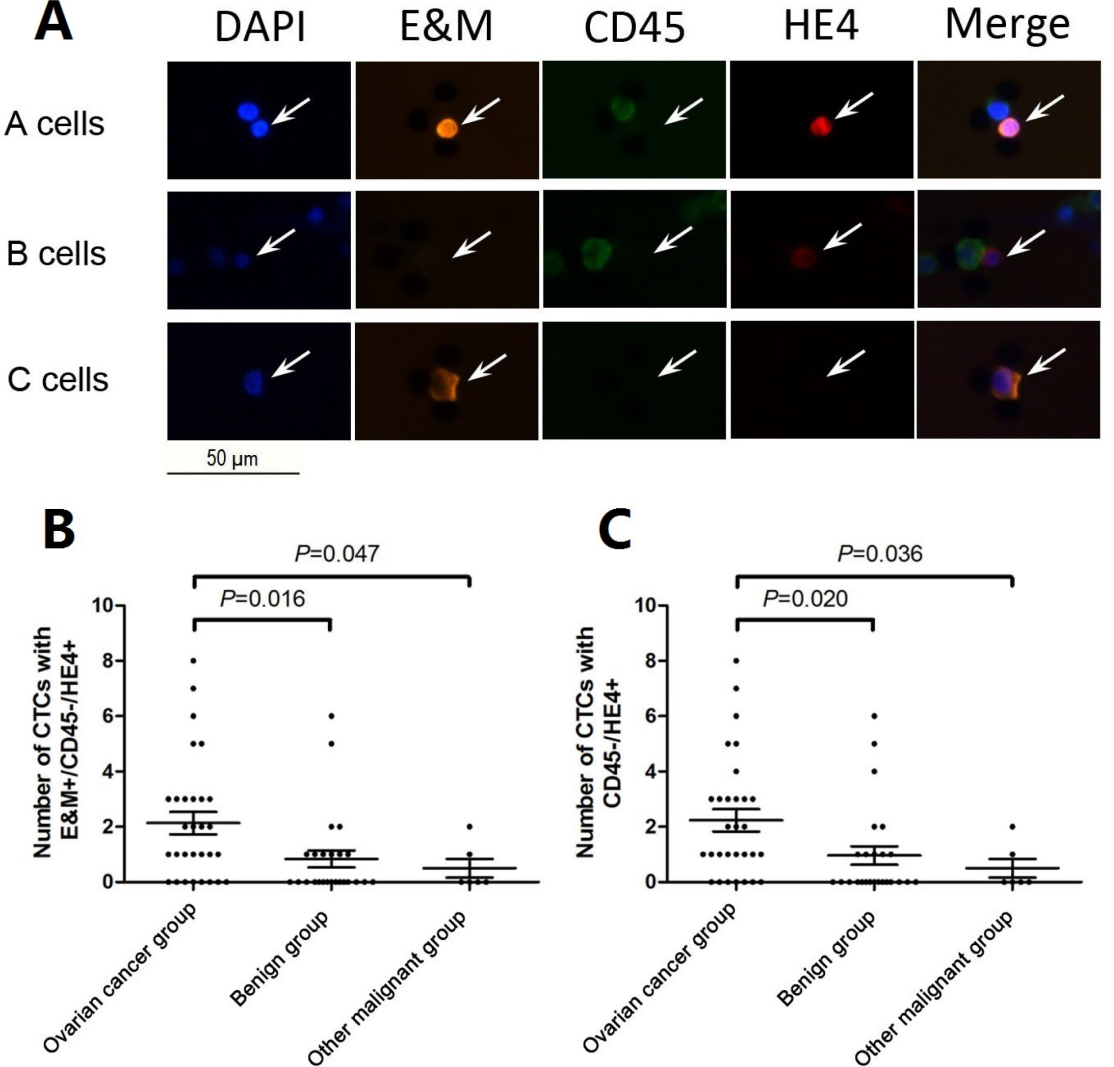
Representative images for three CTC types captured from peripheral blood of patients with suspicious abdominal masses (**A**) Top row: *A cells* with DAPI+/E&M+/CD45-/HE4+; middle row: *B cells* with DAPI+/E&M-/CD45-/HE4+ and bottom row: *C cells* with DAPI+/E&M+/CD45-/HE4-. (**B**) The number of *A cells* with DAPI+/E&M+/CD45-/HE4+ was higher in the ovarian cancer group than the benign (*p =* 0.016) and “other malignant” groups (*p =* 0.047). (**C**) The number of *A + B cells* with DAPI+/CD45-/HE4+ was higher in the ovarian cancer group than the benign (*p =* 0.020) and “other malignant” groups (*p =* 0.036).

We used the following immunofluorescent staining regimes: (DAPI+/E&M+/CD45-/HE4+), (DAPI+/E&M-/CD45-/HE4+), and (DAPI+/E&M+/CD45-/HE4-), to identify the three CTC types, which are referred to hereafter as *A*, *B*, and *C cells*, respectively (Figure 2A). Table 2 lists the mean CTC subtype counts for each patient group. The marker, DAPI+/E&M+/CD45-/HE4+, for identifying *A cells* appeared to be the most effective in immunofluorescent detection of ovarian cancer CTCs. The number of *A cells* in ovarian cancer patients was higher than that in the benign (*p =* 0.016) and “other malignant” groups (*p =* 0.047) (Figure 2B). However, CTC counts did not differ among the tumor subtypes across the three groups (Supplementary Table 1–3). Therefore, we considered combinations of two CTC types. Only numbers of *A* + *B cells* combined (DAPI+/CD45-/HE4+ cells) differed between groups (*p =* 0.020, Figure 2C), while the other two combinations considered (*A* + *C cells*, DAPI+/E&M+/CD45-; and *A* + *B* + *C cells*, DAPI+/CD45-) did not differ (*p* > 0.05, data not shown). Thus, the *A cell* phenotype (DAPI+/E&M+/CD45-/HE4+) could be used to best describe ovarian cancer CTCs. Additionally, all three CTC types were detected in patients with normal and elevated CA125 levels, although their numbers did not differ significantly (*A*: *p =* 0.966, *B*: *p =* 0.635, *C*: *p =* 0.724; Table 3).

**Table 3:** Statistic results of the ROC curve analysis

	Cutoff point	Area under curve	Sensitivity	Specificity	*p*-value
A: In all patients					
A cell	0.5	0.716	0.733	0.630	0.005
B cell	0.5	0.530	0.100	0.963	0.701
C cell	2.5	0.538	0.300	0.852	0.620
A + B cell	0.5	0.715	0.767	0.630	0.005
A + C cell	2.5	0.661	0.533	0.741	0.037
A + B + C cell	3.5	0.534	0.300	0.889	0.660
CA125	98.55	0.691	0.567	0.815	0.013
B: In CA125 normal patients†					
A cell	0.5	0.653	0.778	0.583	0.241
B cell	0.5	0.458	0.000	1.000	0.749
C cell	3.5	0.551	0.333	0.917	0.696
A + B cell	0.5	0.616	0.778	0.583	0.374
A + C cell	11	0.583	0.222	1.000	0.522
A + B + C cell	3.5	0.519	0.333	0.917	0.887
C: In CA125 elevated patients†					
A cell	1.5	0.767	0.571	0.867	0.007
B cell	0.5	0.571	0.143	1.000	0.470
C cell	2.5	0.517	0.286	0.867	0.860
A + B cell	2.5	0.790	0.476	1.000	0.003
A + C cell	2.5	0.689	0.619	0.733	0.056
A + B + C cell	3.0	0.533	0.286	0.867	0.736

*A* cell: DAPI+/E&M+/CD45-/HE4+ cells; *B* cell: DAPI+/E&M-/CD45-/HE4+ cells; *C* cell: DAPI+/E&M+/CD45-/HE4- cells; *A* + *B* cell: DAPI+/CD45-/HE4+ cells; *A* + *C* cell: DAPI+/E&M+/CD45- cells; *A* + *B* + *C* cell: DAPI+/CD45-cells.

^†^CA125 value was estimated as “normal” and “elevated” based on cutoff, 35 U/mL;

### CTCs vs. serum CA125 for screening patients at high risk of ovarian cancer

**Figure 3:**
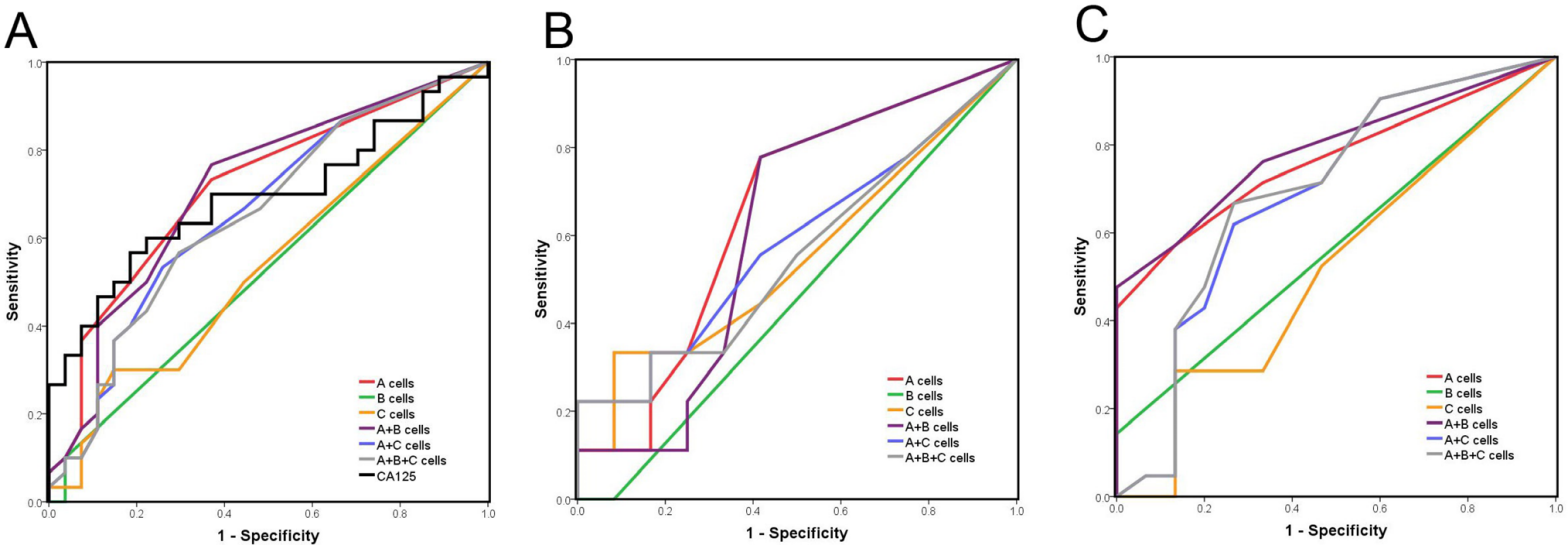
ROC curve analysis in different subgroups **(A**) In total enrolled patients, (**B**) In CA125 normal patients, (**C**) In CA125 elevated patients. Benign and other malignant patients were considered as the control group, cutoff value of CA125 was considered as 35 U/ml.

To compare the diagnostic efficiencies of CTC counts and serum CA125, receiver operating characteristic (ROC) curves were plotted using the benign and “other malignant” groups as the control group (Figure 3A). Taking *A cells* (DAPI+/E&M+/CD45-/HE4+) as the CTC-defining phenotype, we identified cutoff points of ≥0.5/mL for CTCs and ≥98.55 U/mL for CA125 (Table 3A). The area under curve (AUC) for CTCs was larger than that for CA125 (Table 3A). Although specificity was lower for CTCs than CA125, CTC sensitivity was much higher than that of CA125. Subsequently, we considered CTCs and CA125 combined in screening ovarian cancer patients. We analyzed ROC curves for all CTC types detected in CA125 normal (≤35 U/ml) and CA125 elevated (>35 U/ml) patients (Figure 3B and 3C). CTC counts were the same in ovarian cancer cohorts and control cohorts with normal CA125 levels (*p* > 0.05; Table 3B). However, CTC count was an indicator for ovarian cancer in CA125 elevated patients (*p =* 0.007 for *A cells*, Table 3C). Specificity was relatively high (0.867), suggesting that CTC count could be a secondary exclusion criterion in patients with elevated serum CA125.

### CTC count was an independent predictor of ovarian cancer

We examined CTC count as an independent indicator of ovarian cancer using Pearson’s chi-squared test (Table 4). Pearson correlation coefficients for CTC count and other clinicopathological characteristics showed no direct correlations between them, indicating that CTC count could be an independent criterion. However, there was a correlation between ascites and elevated CA125 values (*p =* 0.016; Table 4).

**Table 4:** Correlation analysis of CTCs and other clinicopathological characteristics

Pearson correlation test	CTC count^*^	Lymph node	Peritoneal Metastasis	Ascites	Residual disease	CA125^†^
CTC count^*^		0.795	0.269	0.745	0.507	0.719
Lymph node	0.795		0.604	0.382	0.967	0.204
Peritoneal Metastasis	0.269	0.604		0.395	0.007	0.157
Ascites	0.745	0.382	0.395		0.011	0.016
Residual disease	0.507	0.967	0.007	0.011		0.076
CA125^†^	0.719	0.204	0.157	0.016	0.076	

^*^A cell was used as the criteria for positive and its cutoff value was 0.5/mL as defined in Table 3.

^†^Cutoff value was defined as 35 U/ml.

### HE4+ CTCs were heterogeneous in ovarian cancer patient peripheral blood

**Figure 4:**
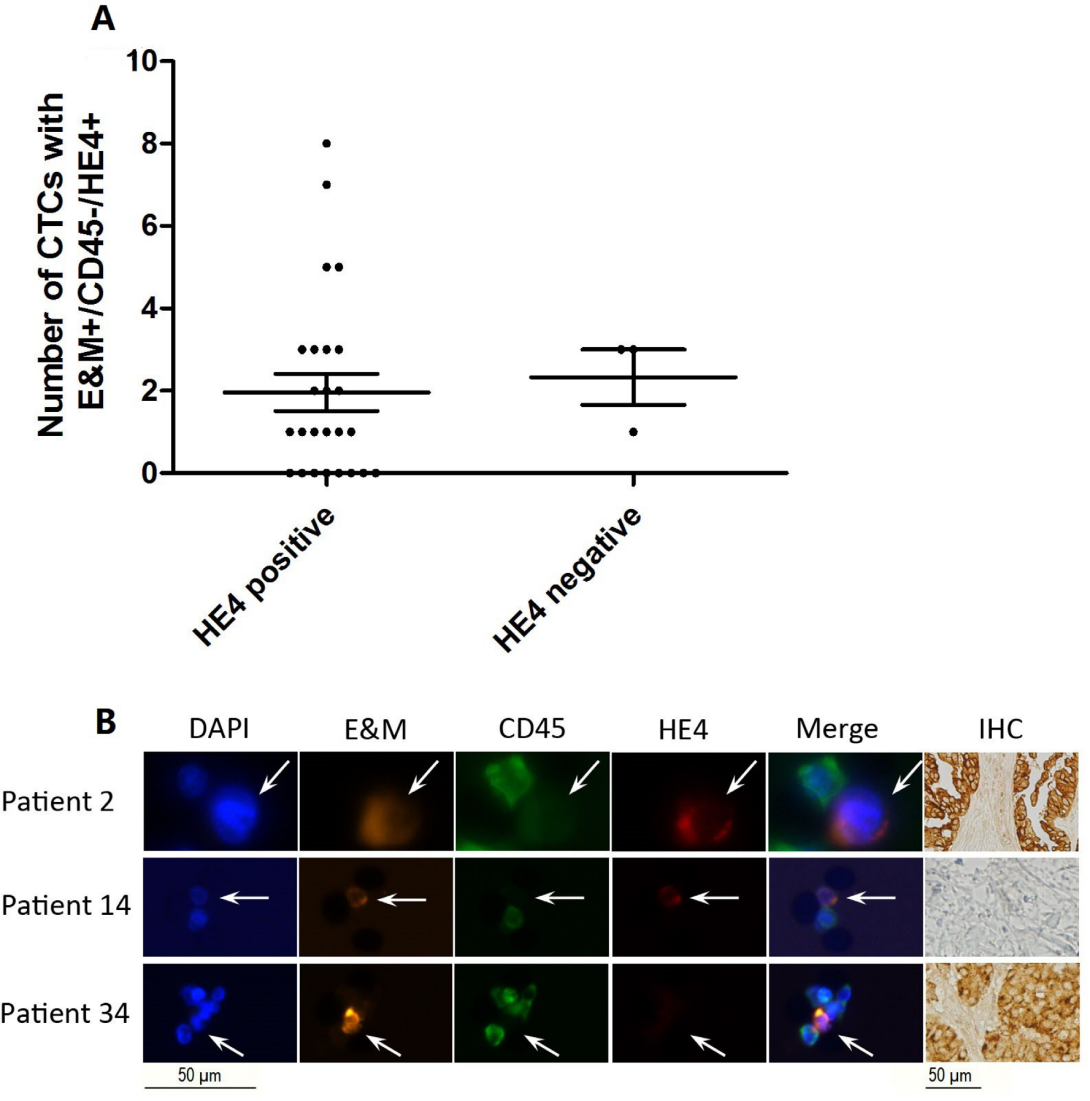
Correlation between HE4 expression in ovarian cancer tissues and peripheral blood CTC count (**A**) There was not significant difference between the number of *A cells* with DAPI+/E&M+/CD45-/HE4+ in HE4 IHC positive patients and that in HE4 IHC negative patients. (**B**) Representative images of DAPI+/E&M+/ CD45-/HE4+ CTCs and HE4+ tissues (top), DAPI+/E&M+/CD45-/HE4+ CTCs and HE4- tissues (middle), and DAPI+/E&M+/CD45-/HE4- cells and HE4+ tissues (bottom).

We performed immunohistochemical (IHC) staining for HE4 in all patients with ovarian cancer. Surprisingly, CTC count in HE4 IHC positive patients was similar to that in HE4 IHC negative patients (*p =* 0.781; Figure 4A). We then assessed CTC counts and paired IHC results for each patient with ovarian cancer. In most HE4 IHC positive patients, HE4+ CTCs were detected in peripheral blood, but in some of these patients, we could only identify DAPI+/E&M+/CD45-/HE4- cells (possibly circulating endothelial cells) (Figure 4B). HE4+ CTCs were also detected in the peripheral blood of HE4 IHC negative patients (Figure 4B), indicating that peripheral HE4+ CTC count might be independent of HE4 expression in the primary carcinoma, and that CTC surface markers might differ compared to the primary site.

## DISCUSSION

Liquid biopsies, including CTC, ctDNA, and exosome assessments, have been considered to be useful methods of monitoring treatment response, assessing the emergence of drug resistance, and quantifying minimal residual disease [35]. Additionally, a new metastasis stage, cM0 (i+), has been added between the traditional M0 and M1 stages for breast and lung cancer as a result of liquid biopsy results by the American Joint Committee on Cancer (AJCC) [36]. The National Comprehensive Cancer Network (NCCN) also incorporates liquid biopsies in their new non-small cell lung cancer guideline [14], and suggests that liquid biopsies should be considered when tissue biopsy is not feasible.

However, much work is still needed to establish CTC detection as a reliable biomarker in ovarian cancer. While CTCs can be detected in ovarian cancer patients, detection rates vary greatly (Table 1), possibly due to patient heterogeneity and the variety of detection methods. The prognostic value of CTCs in ovarian cancer is still in question, as some studies failed to draw significant conclusions.

Epithelial ovarian cancer metastasis is thought to occur at very late stages via direct surface spread [37], and it appears that CTC detection is not useful for early monitoring. A recent study based on the parabiosis model, in which paired mice shared blood but not lymphatic vessels, highlighted hematogenous metastasis as an important mode of ovarian cancer metastasis [10]. Their results also demonstrated a preference for metastasis to the omentum via the hematogenous route, supporting the diagnostic potential of CTCs in patients with suspicious abdominal masses. Thus, accurate capture of CTCs based on size and other biophysical properties, or using markers commonly expressed on the surfaces of these cells [35], could greatly improve cancer detection. EpCAM and cytokeratins are the most widely used surface markers in CTC detection, but detection rates are limited for single markers used alone [27, 28]. Thus, identification methods using multiple markers are the best options for detecting CTCs in ovarian cancer patient peripheral blood.

Serum HE4 is a promising biomarker for discriminating between benign and malignant pelvic masses [38]. HE4 is secreted by cells and can also be detected in the cytoplasm of ovarian cancer cells [39], and may therefore be a useful marker for detecting ovarian cancer CTCs. We stained for HE4 and epithelial or mesenchymal markers (EpCAM, cytokeratins, and vimentin) and found that E&M+/CD45-/HE4+ CTCs had diagnostic significance in patients with pelvic or abdominal masses. However, E&M+/CD45-/HE4- cell numbers did not differ significantly between cancer and control cohorts, at least in part because normal circulating endothelial cells can also exhibit this phenotype. These results indicated that ovarian cancer CTCs express HE4 on the cell surface and that HE4 could be a detection marker for ovarian cancer CTCs.

We also observed that CTC detection results (positive rates) did not exactly match HE4 IHC results in ovarian cancer patients; some HE4 IHC positive patients had negative CTCs, while some HE4 IHC negative patients had positive CTCs. This could be attributed to tumor heterogeneity [9, 35], in that HE4 may not be equably expressed in tumors and CTCs, leading to detection discrepancies between IHC results and CTC counts. Importantly, our study only shows the diagnostic utility of HE4+ CTCs in patients at high risk for ovarian cancer, and we did not attempt to develop a screening test for the general population. While the diagnostic and prognostic utility of CTCs in the general population must be assessed through larger prospective studies, our results support the use of DAPI+/E&M+/CD45-/HE4+ CTC counts for diagnosing ovarian cancer in patients with suspicious pelvic or abdominal masses.

## MATERIALS AND METHODS

### Patients and samples

Sixty-one women admitted to Peking University People’s Hospital between August 2016 and March 2017 and suspected of having ovarian carcinoma were enrolled in the study. Written informed consent was obtained from all patients in accordance with the Declaration of Helsinki. Suspicion of disease was based on clinical examination and ultrasound results. All patients underwent either abdominal or laparoscopic surgery followed by a histological diagnosis. Tumors were classification as IA to IV according to FigureO (2013). Patients receiving neoadjuvant chemotherapy before surgery were excluded. This study was approved by our Institutional Review Board.

Four milliliters of peripheral blood were obtained from each patient for the purpose of CTC detection prior to surgery. Blood samples were drawn in Vacuette EDTA tubes (BD Medical Tech., USA) and centrifuged at 500 g for 5 min at room temperature. Supernatant (plasma) was removed and the remaining blood fraction was processed using a microfluidic device within 4 h.

### Microfluidic chip design and fabrication

The microfluidic chip was adapted from our previous studies on circulating endothelial cells in coronary artery disease patients with angina pectoris [40]. It consisted of eight capture chambers, each 3700 µm (length) × 1036 µm (width) × 25 µm (height). Each chamber had ∼700 capture sites of three different sizes, 8, 10, and 15 µm, to allow smaller erythrocytes and most leukocytes to pass through while retaining larger CTCs. The device had two inlets and one outlet, all equipped with pre-filters (60 µm) to prevent clogging by large debris. One inlet served as the blood sample inlet while the other served as the inlet for reagents needed to perform on-chip immunofluorescence staining. The device was designed using AutoCAD software (Autodesk, USA). We worked with CapitalBio Beijing to fabricate the master mold via photolithography. Briefly, the device pattern was printed on photo film. An 8-inch silicon wafer was spin-coated with a 25 µm-thick layer of SU-8 photoresist (Microchem, USA). The photo film was placed on top of the photoresist and was exposed to UV, followed by post-exposure baking and development to produce a master mold. The microfluidic device was then produced using soft lithography. A 9:1 (weight) mixture of polydimethylsiloxane (PDMS) prepolymer and its crosslinker (Sylgard 184, Dow Corning, USA) was poured onto the master mold and degassed in the vacuum dessicator for 30 min. The PDMS mixture was cured in the oven at 60°C for 4 h. Cured PDMS was then peeled from the master. Holes were punched at designated inlet and outlet ports on the PDMS using a 0.75-mm-diameter puncher (Harris Uni-core, Sigma-Aldrich, USA). The PDMS and a glass slide were cleaned with a piece of Scotch tape, and then oxygen plasma-treated and bonded together. To reinforce bonding, the PDMS device was baked in the oven at 60°C for 20 min.

### Determining chip capture efficiency using a cancer cell line

SKOV3ip1 ovarian cancer cells were cultured in RPMI-1640 media supplemented with 10% FBS and 1% penicillin/streptomycin/amphotericin at 37°C and 5% CO_2_. Before use, cells were stained with CellTracker Red CMTPX dye (Thermo Fisher Scientific, USA) for 30 min and then trypsinized with 0.25% trypsin-EDTA. The capture efficiency of the microfluidic chip was determined by flowing healthy donor blood samples spiked with stained SKOV3ip1 cells (50 cells/ml) through the chip at flowrates of 0.5, 1.0, 1.5, 2.0, and 5.0 ml/h (*n =* 3 per flowrate). Flowrate was controlled using a syringe pump (Longer Pump, China). Captured cells were counted under a microscope. Capture efficiency was evaluated as the percentage of captured cells over spiked cells. To test chip performance at ultralow concentrations of cancer cells, <10 cells were spiked into 1 ml of 1% BSA in 1× PBS buffer. The spiked buffer solution flowed through the chip at 0.5 ml/h. This test was repeated seven times with duplicates.

### Blood processing

One ml of plasma-removed blood was diluted with 1 ml of buffer solution (1% BSA and 8 mM EDTA in 1× PBS). Diluted blood was injected into the microfluidic chip using the syringe pump at 500 µl/h. Larger CTCs were isolated by staggered capture sites with decreasing gap sizes (15–8 µm). On-chip immunofluorescent staining was performed to allow easy cell visualization and enumeration. Briefly, captured cells (CTCs and some leukocytes) were fixed by perfusing 4% paraformaldehyde through the chip for 15 min. Cells were then permeabilized with 0.1% Triton X-100 for 10 min, washed with 1× PBS for 20 min, and blocked with 5% BSA for at least 30 min at room temperature to avoid nonspecific binding of antibodies. A mixture of conjugated antibodies was used: CD45-Alexa Fluor 488 (Invitrogen, USA), EpCAM-phycoerythrin (Abcam, UK), panCK-phycoerythrin (Abcam), vimentin-phycoerythrin (Abcam), CK7/17-phycoerythrin (Novus Biologicals, USA), and HE4-Alexa Fluor 647 (Abcam). DAPI (4′,6-diamidino-2-phenylindole; Thermo Fischer Scientific) was also added to the mixture for nuclear staining. Immunolabeled cells were washed with 1× PBS for at least 30 min. Captured cells were imaged manually using LAS Core microscope imaging software (version 4.4; Leica Microsystems, Germany) and analyzed using ImageJ software (version 1.50i; National Institutes of Health, USA) and Adobe Photoshop CC 2015 software (Adobe Systems, USA).

### ELISA

CA125 was measured in duplicate in each patient serum sample using commercially available enzyme-linked immunosorbent assay (ELISA) kits (E99202Hu, Uscn Life Science, USA) according to the manufacturer’s instructions, with a Multiskan EX plate reader (Thermo Fisher Scientific).

### Immunohistochemistry

IHC assessment of HE4 expression in patient tissues was performed as previously described [41].

### Statistical analysis

Samples from benign tumors and other malignant diseases were established as the control group. Numerical data are presented as means ± standard deviation (range). Two-tailed *t*-test was used to compare the number of CTCs and CA125 levels between two patient subgroups, and ANOVA was used when comparing more than two subgroups. The Mann-Whitney *U* test was performed when sample sizes were insufficient for the *t*-test. ROC curves were used to assess the diagnostic efficiencies of CTC counts and CA125 level. Absolute CA125 levels were interpreted as normal (0, ≤35 U/ml) or elevated (1, >35 U/ml) compared to so-called relative CA125 value. Pearson’s chi-squared test was used to analyze correlations between CTC counts and other clinicopathological characteristics. All statistical analyses were performed using SPSS software (version 19, IBM, USA) and GraphPad Prism software (version 5, GraphPad Software, USA). *p* < 0.05 was considered statistically significant.

## SUPPLEMENTARY MATERIALS TABLES


