# Supplementary material for: Diagnostic value of HE4+ circulating tumor cells in patients with suspicious ovarian cancer
Source: Oncotarget. 2018 Jan 4;9(7):7522–33. doi: 10.18632/oncotarget.23943 (PMC5800921; doi:10.18632/oncotarget.23943)
Supplement: Supplementary file 1 [file oncotarget-09-7522-s001.pdf]

Diagnostic value of HE4+ circulating tumor cells in patients with suspicious ovarian cancer

SUPPLEMENTARY MATERIALS

Supplementary Table 1: CTC counts in different ovarian cancer subtypes

Total	No. of patients (%)	Captured CTC/mL		
	30 (100)	*A* cells (DAPI$^+$/E&M$^+$/CD45$^-$/HE4$^+$)	*B* cells (DAPI$^+$/E&M$^-$/CD45$^-$/HE4$^+$)	*C* cells (DAPI$^+$/E&M$^+$/CD45$^-$/HE4$^-$)
Subtypes (Kruskal-Wallis test)		p = 0.463	p = 0.867	p = 0.098
High-Grade Serous Ovarian Carcinoma	19 (63.3)	1.7 ± 2.1 (0–8)	0.2 ± 0.3 (0–1)	1.5 ± 2.5 (0–10)
Ovarian Clear Cell Carcinoma	5 (16.7)	1.6 ± 1.3 (0–3)	0.0 ± 0.0 (0–0)	5.4 ± 6.0 (0–15)
Low-Grade Serous Ovarian Carcinoma	2 (6.7)	4.0 ± 4.2 (1–7)	0.0 ± 0.0 (0–0)	0.0 ± 0.0 (0–0)
Endometrioid Carcinoma	2 (6.7)	3.0 ± 2.8 (1–5)	0.0 ± 0.0 (0–0)	0.0 ± 0.0 (0–0)
Ovarian Adult Granulosa Cell Tumor	1 (3.3)	3.0	0.0	0.0
Immature Teratoma	1 (3.3)	6.0	0.0	8.0

Supplementary Table 2: CTC counts in different benign tumor subtypes

Total	No. of patients (%)	Captured CTC/mL		
	25 (100)	*A* cells (DAPI$^+$/E&M$^+$/CD45$^-$/HE4$^+$)	*B* cells (DAPI$^+$/E&M$^-$/CD45$^-$/HE4$^+$)	*C* cells (DAPI$^+$/E&M$^+$/CD45$^-$/HE4$^-$)
Subtypes (Kruskal-Wallis test)		p = 0.612	p = 0.922	p = 0. 291
Ovarian Serous Cystadenoma	7 (28)	1.0 ± 1.8 (0–5)	0.4 ± 1.1 (0–3)	1.4 ± 1.8 (0–5)
Ovarian Endometrial Cyst	5 (20)	0.4 ± 0.9 (0–2)	0.0 ± 0.0 (0–0)	2.8 ± 6.3 (0–14)
Ovarian Fibrothecoma	5 (20)	0.6 ± 0.5 (0–1)	0.0 ± 0.0 (0–0)	0.4 ± 0.9 (0–2)
Ovarian Mature Teratoma	3 (12)	0.7 ± 1.2 (0–2)	0.0 ± 0.0 (0–0)	1.0 ± 1.0 (0–2)
Ovarian Mucinous Cystadenoma	2 (8)	0.5 ± 0.7 (0–1)	0.0 ± 0.0 (0–0)	3.5 ± 3.5 (1–6)
Ovarian Fibroma	1 (4)	6.0	0.0	0.0

Pelvic Endometriosis	1 (4)	0.0	0.0	0.0
Ovarian Simple Cyst	1 (4)	0.0	0.0	31.0

Supplementary Table 3: CTC counts in other malignant group

**Total**	**No. of patients (%)**	**Captured CTC/mL**		
	**6 (100)**	***A* cells (DAPI$^+$/E&M$^+$/CD45$^-$/HE4$^+$)**	***B* cells (DAPI$^+$/E&M$^-$/CD45$^-$/HE4$^+$)**	***C* cells (DAPI$^+$/E&M$^+$/CD45$^-$/HE4$^-$)**
Subtypes (Kruskal-Wallis test)		p = 0.636	p = 1.000	p = 0.395
Krukenberg Tumor	2 (33.2)	1.0 ± 1.4 (0–2)	0.0 ± 0.0 (0–0)	1.0 ± 1.4
Retroperitoneal Tumor	1 (16.7)	1.0	0.0	0.0
Rectal Metastatic Carcinoma	1 (16.7)	0.0	0.0	11.0
Colon Metastatic Carcinoma	1 (16.7)	0.0	0.0	3.0
Breast Metastatic Carcinoma	1 (16.7)	0.0	0.0	1.0

Supplementary Table 4: Comparison of the physical characteristics of the patient cohort

	**Ovarian cancer group**	**Benign group**	
Age	57.87 ± 10.98	43.64 ± 15.00	p < 0.001
Weight	62.98 ± 10.82	61.40 ± 9.38	p = 0.568
Height	160.29 ± 5.29	159.70 ± 4.83	p = 0.670
BMI	24.53 ± 4.06	24.02 ± 3.15	p = 0.609
